# Atypical blood glucose response to continuous and interval exercise in a person with type 1 diabetes: a case report

**DOI:** 10.1186/s13256-017-1355-7

**Published:** 2017-06-30

**Authors:** Othmar Moser, Gerhard Tschakert, Alexander Mueller, Werner Groeschl, Thomas R. Pieber, Gerd Koehler, Max L. Eckstein, Richard M. Bracken, Peter Hofmann

**Affiliations:** 1 Exercise Physiology, Training and Training Therapy Research Group, Institute of Sports Sciences, University of Graz, Graz, Austria; 2 Division of Endocrinology and Metabolism, Department of Internal Medicine, Medical University of Graz, Graz, Austria; 3 Diabetes Research Group, Medical School, Swansea University, Swansea, UK; 4 Applied Sport, Technology, Exercise and Medicine Research Centre (A-STEM), College of Engineering, Swansea University, Swansea, UK; 5 Sports Science Laboratory, Institute of Health and Tourism Management, University of Applied Sciences-FH JOANNEUM, Bad Gleichenberg, Austria

**Keywords:** Type 1 diabetes, Exercise, Blood glucose, Hormones

## Abstract

**Background:**

Therapy must be adapted for people with type 1 diabetes to avoid exercise-induced hypoglycemia caused by increased exercise-related glucose uptake into muscles. Therefore, to avoid hypoglycemia, the preexercise short-acting insulin dose must be reduced for safety reasons. We report a case of a man with long-lasting type 1 diabetes in whom no blood glucose decrease during different types of exercise with varying exercise intensities and modes was found, despite physiological hormone responses.

**Case presentation:**

A Caucasian man diagnosed with type 1 diabetes for 24 years performed three different continuous high-intensity interval cycle ergometer exercises as part of a clinical trial (ClinicalTrials.gov identifier NCT02075567). Intensities for both modes of exercises were set at 5% below and 5% above the first lactate turn point and 5% below the second lactate turn point. Short-acting insulin doses were reduced by 25%, 50%, and 75%, respectively. Measurements taken included blood glucose, blood lactate, gas exchange, heart rate, adrenaline, noradrenaline, cortisol, glucagon, and insulin-like growth factor-1. Unexpectedly, no significant blood glucose decreases were observed during all exercise sessions (start *versus* end, 12.97 ± 2.12 *versus* 12.61 ± 2.66 mmol L^−1^, *p* = 0.259). All hormones showed the expected response, dependent on the different intensities and modes of exercises.

**Conclusions:**

People with type 1 diabetes typically experience a decrease in blood glucose levels, particularly during low- and moderate-intensity exercises. In our patient, we clearly found no decline in blood glucose, despite a normal hormone response and no history of any insulin insensitivity. This report indicates that there might be patients for whom the recommended preexercise therapy adaptation to avoid exercise-induced hypoglycemia needs to be questioned because this could increase the risk of severe hyperglycemia and ketosis.

## Background

A precise match in the rates of glucose appearance (Ra) and disappearance (Rd) ensures euglycemia during physical activity and exercise in healthy individuals. In people with type 1 diabetes, hormone responses and the accompanying effects on blood glucose levels are impaired because of autoimmune destruction of insulin-producing β-cells, resulting in a mismatch in Ra and Rd. Shetty *et al*. [1] found an inverted U-shaped relationship between exercise intensity and glucose requirement investigated via glucose infusion rate in a euglycemic clamp. In line with valuable reviews [2, 3], it is suggested to adapt therapy to avoid exercise-induced hypoglycemia. Three possible strategies have been identified to reduce the risk of exercise-induced hypoglycemia: (1) insulin reduction [4], (2) supplementation with extra carbohydrates [5], and (3) the combination of insulin reduction and carbohydrate ingestion [6]. To the best of our knowledge, stable blood glucose levels during different intensities and types of exercise, accompanied by physiological hormone responses, in people with type 1 diabetes have not been reported in any studies.

## Case presentation

Our patient was a Caucasian man with long-lasting type 1 diabetes (age 31 years, body mass index 23.8 kg m^−2^, C-peptide 0.00 nmol L^−1^, glycosylated hemoglobin A1c 60 mmol mol^−1^ [7.6%], diabetes duration 24 years) was switched to insulin degludec (Tresiba®; Novo Nordisk, Bagsvaerd, Denmark) for study purposes (once-daily injection, 19 U/day in the evening) [4]. A run-in period of 4 weeks ensured optimal ultra-long-acting insulin therapy. Before adaptation to insulin degludec, the patient was receiving insulin glargine (Lantus®; Sanofi-Aventis, Paris, France) as a basal routine (twice-daily injections, 5 U in the morning and 18 U in the evening). The patient was treated with insulin lispro (Humalog®; Eli Lilly Australia, West Ryde, Australia) with a carbohydrate factor of 1 U of insulin lispro per 12 g of carbohydrates; a correction factor of 1 U of insulin lispro lowers blood glucose by 2.8 mmol L^−1^.

The patient showed normal signs for the respiratory, cardiovascular, and gastrointestinal systems. The results of skin and lymph node palpation examinations were normal. No signs of neuropathy were found, and the patient was free of any comorbidities. The results of urine microalbumin and retinopathy screening were negative. The patient’s blood pressure after 5 minutes of resting was 118 mmHg (systolic)/74 mmHg (diastolic) at the screening visit. Laboratory assessment revealed normal results: C-reactive protein 5.71 nmol L^−1^, aspartate aminotransferase 0.4 μkat L^−1^, alanine aminotransferase 0.28 μkat L^−1^, alkaline phosphatase 0.72 μkat L^−1^, γ-glutamyltransferase 0.25 μkat L^−1^, albumin 61.4%, natrium 137 mmol L^−1^, potassium 4.4 mmol L^−1^, magnesium 0.77 mmol L^−1^, urea 12.14 mmol L^−1^, creatinine 83.88 μmol L^−1^, glomerular filtration rate 1.5 ml s^−1^ m^−2^, and uric acid 392.6 μmol L^−1^.

### Exercise testing

The patient performed a maximum incremental exercise test on a cycle ergometer to determine exercise intensities for the continuous and interval exercise tests. The first lactate turn point (LTP_1_) and the second lactate turn point (LTP_2_) were identified by a linear regression break point analysis for the lactate concentration to power output curve. Low (L), moderate (M), and heavy (H) workload intensities for the continuous and interval exercises were set at 5% of the maximum power output (P_max_) from the incremental exercise test below (L) and above (M) LTP_1_ and below (H) LTP_2_. Intervals were set at the P_max_ from the incremental exercise test for 20 seconds. For L, recovery duration (*t*_rec_) was 120 seconds (work to rest ratio 1:6); for M, *t*_rec_ was 60 seconds (work to rest ratio 1:3); and for H, *t*_rec_ was 20 seconds (work to rest ratio 1:1). All exercises were performed for 30 minutes at the target workload at the same time of day, and each exercise session was separated by 1 week. The continuous and interval exercises were matched for total duration and mean workload [4]. Short-acting insulin dose was reduced by 40% for the incremental exercise test, 25% for L, 50% for M, and 75% for H, 4 hours before the start of each exercise session. Additional details about this study were presented previously [4].

### Measurements

Blood glucose and blood lactate concentrations were determined by taking capillary blood samples from the earlobe at all tests while the patient was at rest and during warmup, exercise, and recovery. Capillary blood samples were analyzed by means of an enzymatic method with amperometric monitoring (Biosen S-line®; EKF Diagnostics, Barleben, Germany). Gas exchange data were collected continuously during all tests, and 5-second average data were used for analyses (ZAN 600; nSpire Health, Oberthulba, Germany). The patient’s heart rate was measured beat-to-beat during all tests, and 5-second average data were used for calculations (PE 4000; Polar Electro Finland Oy, Kempele, Finland). Adrenaline, noradrenaline, cortisol, insulin-like growth factor 1, and glucagon levels were measured using blood samples obtained from the cubital vein before the start of exercise, after 15 minutes of exercise, and at the end of each 30 minutes of continuous and interval exercise testing.

### Findings

**Fig. 1:**
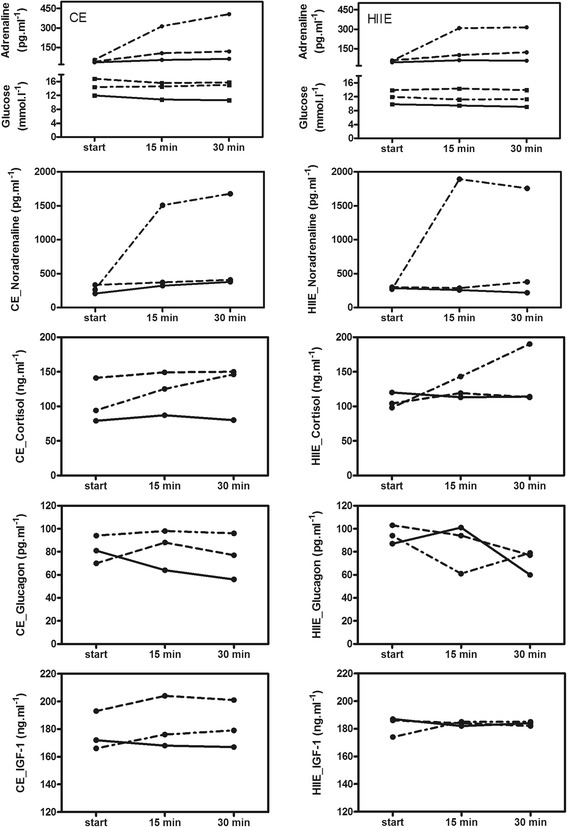
Adrenaline, glucose, noradrenaline, cortisol, glucagon, and insulin-like growth factor 1 responses for the three different exercise intensities (*solid line* = low workload, *dashed line* = moderate workload, *dashed-dotted line* = heavy workload) for continuous exercise and high-intensity interval exercise in a single patient with type 1 diabetes exhibiting an atypical blood glucose time course during exercise. Accompanying blood glucose levels for continuous exercise and high-intensity interval exercise are given for adrenaline responses in the first segment (*solid line* = low workload, *dashed line* = moderate workload, *dashed-dotted line* = heavy workload). *CE* Continuous exercise, *HIIE* High-intensity interval exercise

The incremental exercise test resulted in a maximum oxygen uptake of 57 ml kg^−1^ minute^−1^ and a P_max_ of 300 W. Absolute values of power output were 86 W at LTP_1_ and 205 W at LTP_2_, respectively. Mean exercise intensities prescribed in relation to LTP_1_ and LTP_2_, for both the continuous and interval exercises, corresponded to 71 W (24% of P_max_) for L, 101 W (34% of P_max_) for M, and 190 W (63% of P_max_) for H. Short-acting insulin reductions at the start of the exercises resulted in blood glucose concentrations of 11.97 mmol L ^−1^ (L), 15.75 mmol L ^−1^ (M), and 14.43 mmol L ^−1^ (H) for the continuous exercises tests, as well as 9.82 mmol L ^−1^ (L), 13.87 mmol L ^−1^ (M), and 11.95 mmol L ^−1^ (H) for the interval exercise tests, respectively. Delta blood glucose levels (difference between start and end of the exercise) for the continuous exercise tests were 1.38 mmol L ^−1^ (L), 0.00 mmol L ^−1^ (M) and −0.57 mmol L ^−1^ (H), as well as 0.73 mmol L ^−1^ (L), −0.03 mmol L ^−1^ (M), and 0.65 mmol L ^−1^ (H), for the interval exercise tests. Normal hormone responses were found, as displayed in Fig. 1. Furthermore, normal responses in lactate, respiratory data, and heart rate were observed, as shown in Table 1.

**Table 1:** Lactate, respiratory exchange ratio, and heart rate responses during continuous exercise and high-intensity interval exercise of low, moderate, and high intensities

	Low intensity	Moderate intensity	High intensity
Lactate			
CE, mmol L^−1^	0.59 ± 0.11	1.32 ± 0.19	4.52 ± 0.70
HIIE, mmol L^−1^	1.44 ± 0.45	2.57 ± 0.55	5.11 ± 1.37
RER			
CE	0.81 ± 0.03	0.88 ± 0.03	0.91 ± 0.03
HIIE	0.93 ± 0.08	0.94 ± 0.07	0.95 ± 0.04
Heart rate			
CE, beats minute^−1^	99 ± 3	111 ± 5	164 ± 8
HIIE, beats minute^−1^	93 ± 16	118 ± 12	165 ± 11

*Abbreviations: CE* Continuous exercise, *HIIE* High-intensity interval exercise, *RER* Respiratory exchange ratio

## Discussion

As stated by Shetty *et al*. [1], blood glucose generally drops during low- and moderate-intensity exercise in people with type 1 diabetes, but it may even increase during and after high-intensity exercise [7]. Adrenaline and noradrenaline have been shown to be the main regulators to target for avoiding blood glucose decreases during high-intensity exercise due to increased rates of glycogen breakdown. Contrarily, our laboratory previously found a decrease in blood glucose concentration in all intensity domains of continuous and high-intensity interval exercise, which was found to be significantly related to exercise intensity [8].

Paradoxically, in our patient, during neither low- nor moderate- or high-intensity exercise did blood glucose decrease as expected, independently of the mode of exercise applied, despite other markers being found to be within normal limits. Although the patient’s catecholamine levels were comparable to the whole group investigated [4] and did not reveal exaggerated counteraction, no changes in blood glucose concentrations were observed. At first sight, no plausible causes for such a paradoxical response can be suggested, because the person injected his regular basal insulin dose, did not ingest supplemental carbohydrates, and did not have any known history of insulin insensitivity.

One hypothetical explanation might be impaired glucose transporter type 4 (GLUT-4) translocation during exercise. Especially during low-intensity exercise, a significant increase in myosin heavy chain, type I, has been reported [9], which enhances muscular glucose uptake, accompanied by a decrease in blood glucose concentration in type 1 diabetes. Even so, a detailed mechanism of GLUT-4 response to muscle contraction is not yet fully understood, but it is assumed that upstream signaling pathways that lead to GLUT-4 translocation include 5′-adenosine monophosphate-activated protein kinase, Ca^2+^/calmodulin-dependent protein kinase II, and nitric oxide synthase [9]. If one of these signaling molecules is inhibited, this could influence the blood glucose response during physical activity and exercise.

To the best of our knowledge, this is the first report of no blood glucose decrease during different types of exercise with varying exercise intensities and modes in a man with long-lasting type 1 diabetes, despite physiologically normal hormone responses were found. Exaggerated insulin dose reductions can cause hyperglycemia and may lead to development of ketosis during physical activity. Especially in people with type 1 diabetes who are performing high-intensity exercises on a regular basis (for example, sport games, interval training, competitive sports), stable or increasing blood glucose responses during exercise might raise the risk of life-threatening ketosis.

## Conclusions

For our patient, who did not respond even to low- and moderate-intensity exercise, we discouraged preexercise insulin dose reductions to avoid severe hyperglycemia and to minimize any negative impact of regular periexercise hyperglycemia on long-term glycemic control. From a clinical point of view, the finding in our study highlights the need to perform different types of exercise tests (incremental exercise test, continuous and interval exercises) in physically active people with type 1 diabetes. Depending on individual sports, this should be performed to avoid drastic hyperglycemic blood glucose excursions because there might be glucose nonresponders to low- and moderate-intensity exercise.

## Abbreviations

CE: Continuous exercise
GLUT-4: Glucose transporter type 4
H: Heavy workload
HIIE: High-intensity interval exercise
L: Low workload
LTP_1_: First lactate turn point
LTP_2_: Second lactate turn point
M: Moderate workload
P_max_: Maximum power output
Ra: Rate of glucose appearance
Rd: Rate of glucose disappearance
RER: Respiratory exchange ratio
*t*_rec_: Recovery duration
VO_2max_: Maximum oxygen uptake

## References

[1] Shetty VB, Fournier PA, Davey RJ, Retterath AJ, Paramalingam N, Roby HC, *et al.* Effect of exercise intensity on glucose requirements to maintain euglycemia during exercise in type 1 diabetes. J Clin Endocrinol Metab. 2016;101:972–80. 10.1210/jc.2015-4026 26765581

[2] Galassetti P, Riddell MC. Exercise and type 1 diabetes (T1DM). Compr Physiol. 2013;3:1309–36. 10.1002/cphy.c110040 23897688

[3] Pinsker JE, Kraus A, Gianferante D, Schoenberg BE, Singh SK, Ortiz H, *et al.* Techniques for exercise preparation and management in adults with type 1 diabetes. Can J Diabetes. 2016;40:503–8. 10.1016/j.jcjd.2016.04.010 27212045

[4] Moser O, Tschakert G, Mueller A, Groeschl W, Pieber TR, Obermayer-Pietsch B, *et al.* Effects of high-intensity interval exercise versus moderate continuous exercise on glucose homeostasis and hormone response in patients with type 1 diabetes mellitus using novel ultra-long-acting insulin. PLoS One. 2015;10:e0136489. 10.1371/journal.pone.0136489 PMC4552855 26317981

[5] Francescato MP, Carrato S. Management of exercise-induced glycemic imbalances in type 1 diabetes. Curr Diabetes Rev. 2011;7:253–63. 10.2174/157339911796397875 21682691

[6] West DJ, Stephens JW, Bain SC, Kilduff LP, Luzio S, Still R, *et al.* A combined insulin reduction and carbohydrate feeding strategy 30 min before running best preserves blood glucose concentration after exercise through improved fuel oxidation in type 1 diabetes mellitus. J Sports Sci. 2011;29:279–89. 10.1080/02640414.2010.531753 21154013

[7] Riddell MC, Gallen IW, Smart CE, Taplin CE, Adolfsson P, Lumb AN, *et al.* Exercise management in type 1 diabetes: a consensus statement. Lancet Diabetes Endocrinol. 2017;5:377–90. 10.1016/S2213-8587(17)30014-1 28126459

[8] Moser O, Tschakert G, Mueller A, Groeschl W, Hofmann P, Pieber TR,* et al.* Short-acting insulin reduction strategies before continuous ergometer exercises in patients with type 1 diabetes mellitus. Asian J Sports Med. 2017;8:e42160.

[9] Richter EA, Hargreaves M. Exercise, GLUT4, and skeletal muscle glucose uptake. Physiol Rev. 2013;93:993–1017. 10.1152/physrev.00038.2012 23899560

